# DZIP1 regulates mammalian cardiac valve development through a Cby1-β-catenin mechanism

**DOI:** 10.1002/dvdy.342

**Published:** 2021-04-09

**Authors:** Lilong Guo, Tyler Beck, Diana Fulmer, Sandra Ramos-Ortiz, Janiece Glover, Christina Wang, Kelsey Moore, Cortney Gensemer, Jordan Morningstar, Reece Moore, Jean-Jacques Schott, Thierry Le Tourneau, Natalie Koren, Russell A. Norris

**Affiliations:** 1 Department of Regenerative Medicine and Cell Biology, Medical University of South Carolina, Charleston, South Carolina; 2 Inserm U1087, Institut du Thorax, University Hospital, Nantes, France

**Keywords:** b-catenin, cilia, DZIP1, valve development

## Abstract

**Background:**

Mitral valve prolapse (MVP) is a common and progressive cardiovascular disease with developmental origins. How developmental errors contribute to disease pathogenesis are not well understood.

**Results:**

A multimeric complex was identified that consists of the MVP gene Dzip1, Cby1, and β-catenin. Co-expression during valve development revealed overlap at the basal body of the primary cilia. Biochemical studies revealed a DZIP1 peptide required for stabilization of the complex and suppression of β-catenin activities. Decoy peptides generated against this interaction motif altered nuclear vs cytosolic levels of β-catenin with effects on transcriptional activity. A mutation within this domain was identified in a family with inherited non-syndromic MVP. This novel mutation and our previously identified *DZIP1*^*S24R*^ variant resulted in reduced DZIP1 and CBY1 stability and increased β-catenin activities. The β-catenin target gene, MMP2 was up-regulated in the *Dzip1*^*S14R/+*^ valves and correlated with loss of collagenous ECM matrix and myxomatous phenotype.

**Conclusion:**

Dzip1 functions to restrain β-catenin signaling through a CBY1 linker during cardiac development. Loss of these interactions results in increased nuclear β-catenin/Lef1 and excess MMP2 production, which correlates with developmental and postnatal changes in ECM and generation of a myxomatous phenotype.

## 1 | INTRODUCTION

Mitral valve prolapse (MVP) affects 2% to 3% of the general population and is associated with secondary co-morbidities such as arrhythmias, heart failure, and sudden cardiac death.^1,2^ MVP is characterized as the billowing of one or both leaflets above the level of the mitral annulus during cardiac systole. Structural changes of the valve result in an inability for the valve to be mechanically proficient during the cardiac cycle. These changes are characterized by alterations in the amount and types of extracellular matrix (ECM) present within the valves. For example, increased proteoglycan and collagen deposition are evident with fragmented collagen and elastin being present. The loss of normal zonal boundaries is evident with expansion of proteoglycans throughout the valve layers.^3^ Valve interstitial cells (VICs) are thought to contribute to valve degeneration by becoming activated into myofibroblasts which undergo hyperplasia and produce excess ECM and growth factor ligands.^4–8^ Over time, the valve becomes thickened and floppy which results in incompetence, prolapse, and mitral regurgitation. Surgery is the only curative option for patients with severe MVP.^9,10^

To date, the molecular and cellular causes of MVP are poorly understood. Recent genetic studies have revealed a role for primary cilia during valvulogenesis, indicating a potential unifying pathway for disease initiation.^11–15^ Recently, mutations in the cilia gene *DZIP1* were reported in multiple families with inherited, autosomal dominant non-syndromic MVP.^14^ DZIP1 is a zinc finger protein localized to the basal body and nucleus and can regulate various downstream pathways involved in hedgehog and Wnt/β-catenin signaling.^16–18^ However, how Dzip1 mutations result in MVP is unknown.

To investigate the mechanism of DZIP1 in MVP, we identified Chibby-1 (CBY1) a binding partner of DZIP1 thorough proteomics analyses. CBY1 is not only a ciliary gene essential for both motile and primary cilia assembly,^19–21^ but has also been shown to be a weak β-catenin antagonist by directly interacting with β-catenin to affect nuclear vs cytoplasmic shuttling of β-catenin.^22^ In this study, we report that a protein complex consisting of DZIP1-CBY1 and β-catenin are required for valve morphogenesis and disruption of their interactions cause valve defects and alterations in ECM production that progress to myxomatous degeneration. Additionally, identification of a multigenerational family with a rare, potentially damaging mutation within the DZIP1-CBY1 interaction domain further support this pathway as causative in patients with MVP.

## 2 | RESULTS

### 2.1 | CBY1 Interacts and colocalizes with DZIP1 in developing mitral valves

**FIGURE 1:**
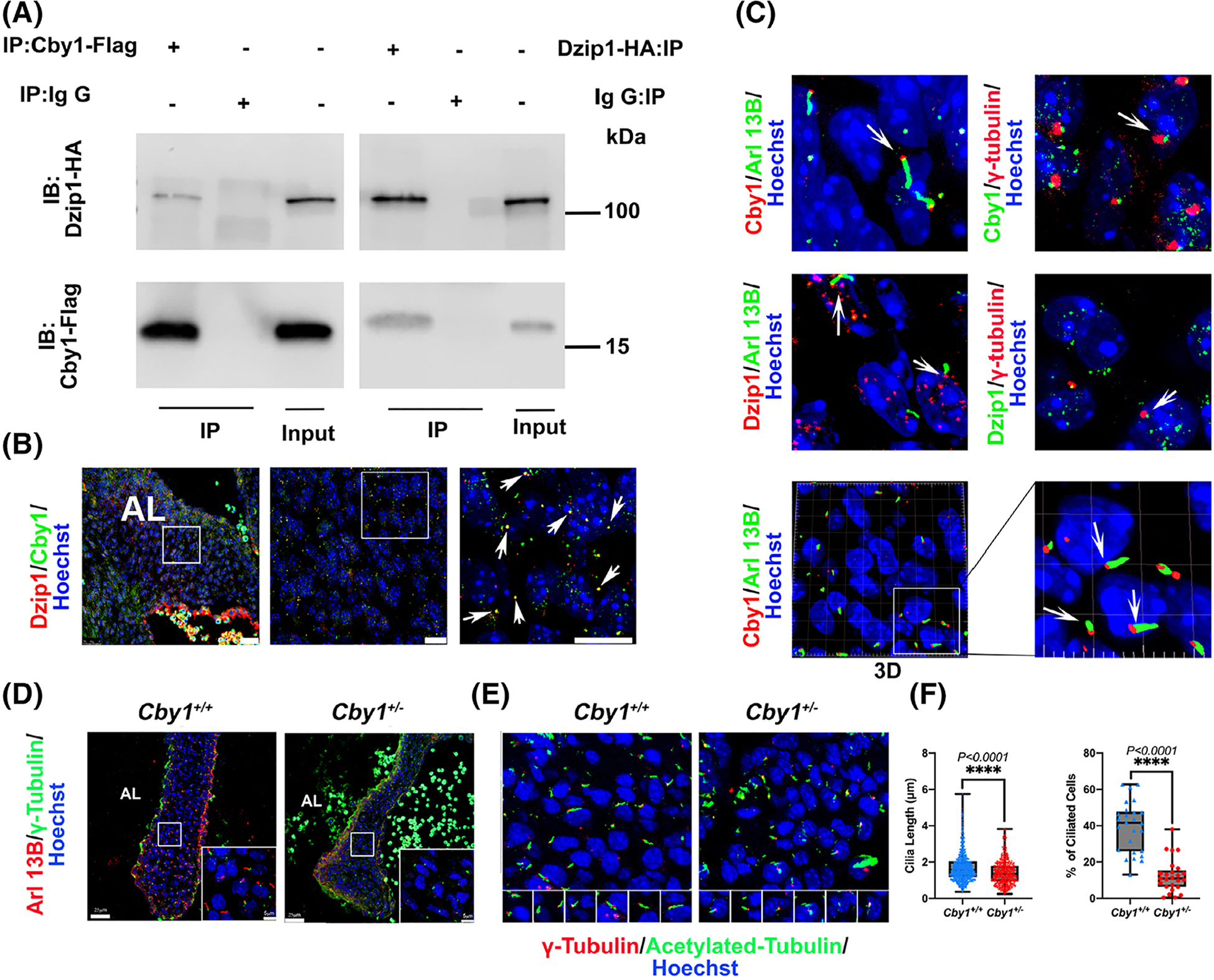
CBY1 interacts with DZIP1 and localizes to the basal body in developing mitral valves. A, Co-Immunoprecipitation (IP) and immunoblotting (IB) analysis of DZIP1 with CBY1 in HEK293T whole-cell lysis showing an interaction between these two proteins. B, IHC for DZIP1(red), CBY1(green) and nuclei (Hoechst-blue) in E13.5 mitral valves. Arrows show expression overlap of DZIP1 and CBY1. Left: anterior leaflet (AL) of the mitral valves, scale bar = 25 μm, Middle: higher magnification of the left image boxed area, scale bar = 10 μm; Right: higher magnification of boxed area of middle image, scale bar = 10 μm. C, IHC for Arl13B, γ-tubulin, CBY1, DZIP1 and nuclei (Hoechst-blue) in E13.5 mitral valves. Left panel: Arl13B(green), DZIP1/CBY1(red); Right panel: γ-tubulin(red), DZIP1/CBY1(green). Bottom panel: 3D reconstruction of CBY1(red) and Arl13B(green). D, Representative images of *Cby1*^+/−^ and *Cby1*^+/+^ P0 anterior mitral leaflet immunostained for Arl13B (red), γ-tubulin (green) and nuclei counterstained with hoechst (blue) compared to littermate control. E, *Cby1*^+/−^ P0 anterior leaflet immunostained for acetylated-tubulin (red), γ-tubulin (red) and nuclei counterstained with hoechst (blue) compared to littermate control. F, Quantification of primary cilia length and percentage of ciliated cells for *Cby1*^+/−^ P0 anterior leaflet compared to littermate control. A statistically significant decrease in percentage of ciliated cells and length of primary cilia were evident in *Cby1*^+/−^ mitral valves. Scale bar = 25 μm, scale bar of boxed area = 5 μm

To identify direct binding partners for DZIP1 in the heart, yeast two-hybrid screens were performed where full length DZIP1 was used as the bait. Of note, 113 million clones from a human embryonic and adult heart library were screened. CBY1 was identified as one of the most prevalent interactors with 14 total clones being identified from the screen. Co-immunoprecipitations (Co-IPs) confirmed an interaction between DZIP1 and CBY1 in HEK293T cells (Figure 1A). Co-expression of CBY1 and DZIP1 was analyzed by immunohistochemistry (IHC) at E13.5 and revealed overlap of Dzip1 with Cby1 at the basal body of primary cilia in most valve mesenchymal cells that were visualized (Figure 1B,C). Whereas Dzip1 is observed both at the basal body and in the nucleus as we previously reported,^14^ Cby1 is enriched at the basal body. Within the basal body, the pattern of both Dzip1 and Cby1 appear similar and stain only a small fraction of this subcellular structure, suggesting a function within a discrete portion of the basal body. Areas in which the two proteins did not co-localize was also evident, possibly representing unique subcellular expression domains or various snapshots of expression within shuttling complexes.

We recently reported that Dzip1 is required for ciliogenesis in the mitral valves.^14^ Due to our findings that Cby1 interacts with Dzip1 in the mitral valves, we tested whether the Cby1 was similarly required for ciliogenesis. Cilia length and percentage of ciliated cells on *Cby1*^+/−^ and wild-type P0 mouse anterior leaflets were quantified (Figure 1D–F). Much like our findings in Dzip1 deficient animal models, cilia length and percentage of ciliated cells are significantly decreased in *Cby1*^+/−^ valves compared with wild-type mitral leaflets suggesting shared molecular pathways between Dzip1 and Cby1 in regulating ciliogenesis.

### 2.2 | Mapping of the DZIP1-CBY1 interaction domain

**FIGURE 2:**
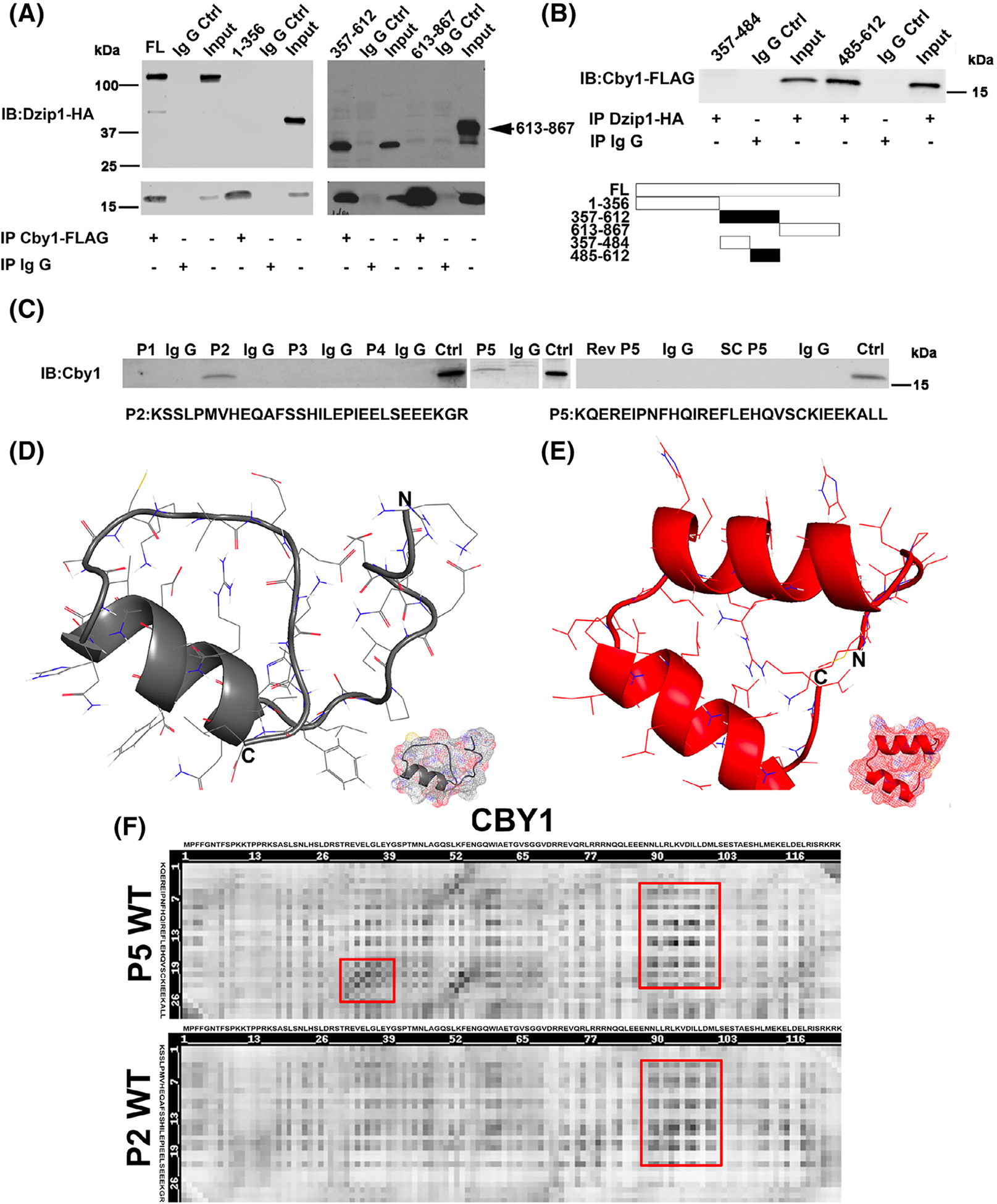
Mapping of DZIP1-CBY1 interaction domain. A, Co-immunoprecipitation (Co-IP) and immunoblot (IB) of CBY1 with DZIP1 truncated constructs in HEK 293T showing region 357–612 harbors an interaction domain. FL, full length DZIP1, numbers represent DZIP1 amino acids that are encoded by the DNA in the expression constructs. Ctrl, control. B, Co-IP and IB analysis showing an interaction within the 485–612 region of DZIP1. C, Co-IP and IB analysis of CBY1 with biotinylated peptides. Two peptides (P2: amino acids 531–560 and P5: amino acids 564–593) were positive for binding with CBY1. Scrambled (SC P5) and Reverse (Rev P5) peptides for P5 served as negative controls. D and E, Computational modeling of the two peptides: P2 (D) and P5 (E). Modeling was generated through RaptorX. F, Heatmap analysis of predicted peptide binding residues within CBY1 across P2 and P5. Red box, similar binding residues within CBY1 between P2 and P5

Various amino and carboxyl-termini deletion constructs for DZIP1 were generated and expressed in HEK293T cells followed by Co-IP in order to identify CBY1-interaction motifs. Constructs expressing just the N-terminal 356 amino acids (1–356) failed to interact with CBY1. Likewise, constructs only expressing the amino acids 613-to the C-terminus (613–867) were unable to interact with CBY1. However, amino acids 357 to 612 revealed a positive interaction between DZIP1 and CBY1 (Figure 2A). This region was further dissected into two polypeptide stretches and tested by Co-IP. As shown in Figure 2B, only region 485 to 612 harbors the DZIP1-CBY1 interaction motif. Fine mapping of this region was performed by generating overlapping biotinylated polypeptides and tested by Co-IP (Figure 2C). Within this region we identified a bipartite interaction motif in which two unique peptides (amino acids 531–560 and 564–593) were capable of independently interacting with CBY1 (P2: 531–560 and P5:564–593). Scrambled and reverse peptides for P5 served as negative controls and failed to show an interaction as expected. Computational modeling of the peptides revealed similarities between these two peptides in conformation indicating a potential likelihood of interacting with overlapping/similar residues on CBY1 (Figure 2D,E). Heatmap analyses showed that the P2 and P5 peptides are predicted to interact within similar regions of the CBY1 protein indicating that structurally, these two regions of DZIP1 are similarly oriented in three-dimensional space (Figure 2F).

### 2.3 | DZIP1 peptide interacts with CBY1 and β-catenin

**FIGURE 3:**
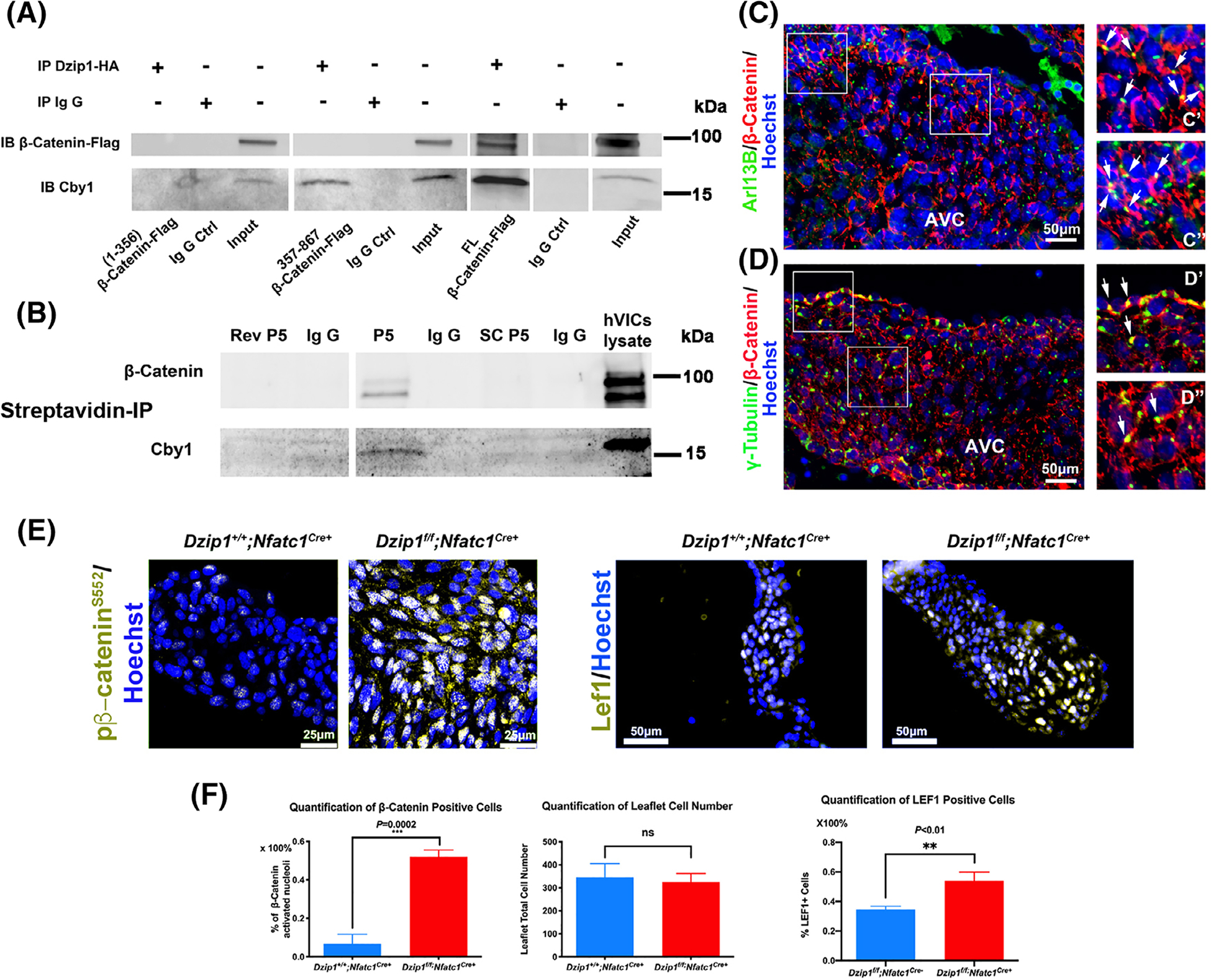
DZIP1 interacts with β-catenin through CBY1. A, Co-IP and IB analysis of β-catenin with DZIP1 in HEK293T whole-cell lysis. Constructs are denoted below the Western blot. Full length (FL) Dzip1 can interact with Cby1 and β-catenin. Numbers represent DZIP1 amino acids that are encoded by the DNA in the expression constructs. Ctrl, control. B, Co-IP and IB of biotinylated P5 (peptide #5) showing an interaction with Cby1 and β-catenin but not with the reverse (rev.) or scrambled (SC) P5 peptides. C, IHC for β-catenin (red), Arl13B (green) and nuclei (Hoechst-blue) in 13.5 mitral valves. C′ & C″, Higher magnification of boxed region in C showing expression at the base of primary cilia. D, IHC for β-catenin (red), γ-tubulin (green) and nuclei (Hoechst-blue) in 13.5 mitral valves. D′ & D″, Higher magnification of boxed region in D showing expression at the basal body. E, Left: P0 *Dzip1*^*f/f*^*;Nfatc1*^*Cre+*^ murine mitral valves immunostained for pβ-catenin^S552^ (gold) and nuclei counterstained with hoechst (blue) compared to littermate control. Right: P0 murine mitral valves immunostained for Lef1 (gold) and nuclei counterstained with hoechst (blue) compared to littermate control. F, Left: quantification of pβ-catenin^S552 +^ cell percentage; middle: quantification of anterior leaflet cell number; right: quantification of Lef1^+^ cell percentage. Data are means ± SD, unpaired two-tailed Student’s *t*-test. (n = 3/genotype, ***P = .0002, **P < .01, ns, non-significant)

As Cby1 was previously shown to directly interact with β-catenin,^23^ we used Co-IP experiments to test whether Dzip1 was capable of a β-catenin interaction through a CBY1 linker moiety. As shown in Figure 3A, full length (FL) Dzip1 was able to interact with both CBY1 and β-catenin. When we tested the amino end (1–356) DZIP1 expression construct, no interaction with CBY1 or β-catenin was observed, as expected. When the C-terminal 357 to 867 amino acids of DZIP1 were tested, a CBY1 interaction was detected, but β-catenin was not evident in the IP reaction. This paradoxical observation indicated that upon CBY1 binding to DZIP1, the amino end of DZIP1 may be important in stabilizing the β-catenin interaction. It also remains possible that in the absence of the amino end of DZIP1, the 357 to 867 DZIP1 polypeptide folds around CBY1, encasing the protein, hindering interaction with other proteins. To test this hypothesis, we assayed whether the minimal DZIP1-CBY1 interaction motif (peptide 5), was sufficient to pull down the entire complex. As shown in Figure 3B, peptide 5 (P5) was able to coimmunoprecipitate both CBY1 and β-catenin in cultured human VICs. Subsequent immunohistochemistry confirmed that β-catenin protein is found both at the level of the cell membrane as well as at the basal body in developing murine mitral valves at E13.5. Although the expression of β-catenin is obviously more widespread on the cell membranes, there is coincident tempero-spatial expression with Dzip1 and Cby1 at the base of the primary cilia (Figure 3C,D).

### 2.4 | Loss of Dzip1 results in increased nuclear β-catenin and Lef1

**FIGURE 4:**
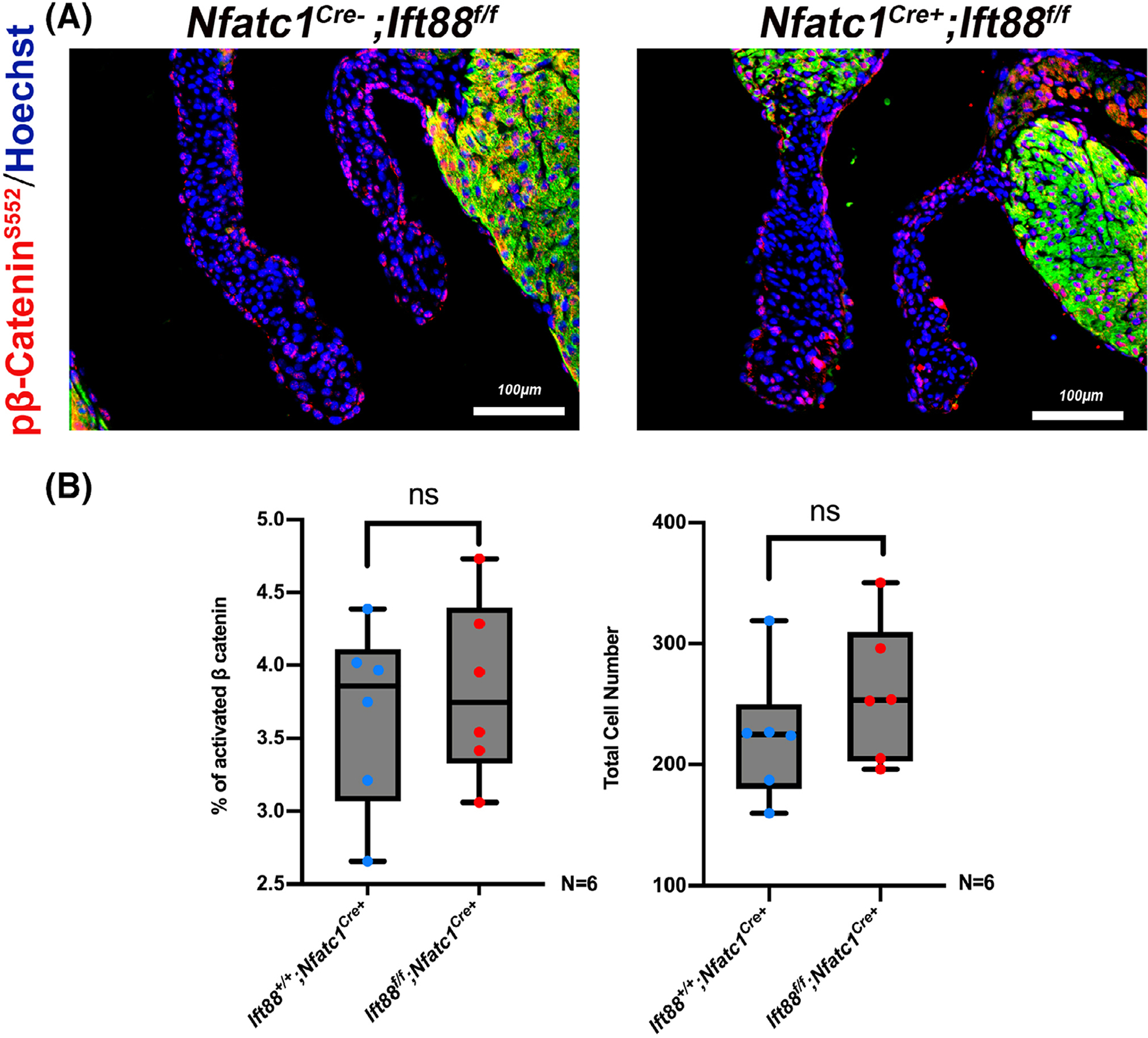
Regulation of β-catenin is independent of primary cilia. A, Representative images of *NfatC1*^*Cre+*^*; Ift88*^*f/f*^ P0 murine mitraI valves immunostained for pβ-catenin^S552^ (red), MF20 (green) and nuclei counterstained with hoechst (blue) compared to littermate controls (*NfatC1*^*Cre*−^*;Ift88*^*f/f*^). B, Quantification of *NfatC1*^*Cre+*^*; Ift88*^*f/f*^ P0 murine anterior leaflet for pβ-catenin^S552 +^ cell percentage and total cell number compared to littermate control. Data are means ± SD, unpaired two-tailed Student’s *t*-test. (n = 6/genotype, ns, non-significant)

An interaction between DZIP1, CBY1 and β-catenin combined with our co-expression studies showing Cby1 is only present at the basal body, suggested that the DZIP1-CBY1 complex functions to sequester β-catenin. To test this hypothesis, we analyzed if loss of Dzip1 in the mitral valves would release this inhibition. As shown in Figure 3E, F, conditional deletion of Dzip1 from endocardium and endocardial derived mesenchyme (*Dzip1*^*f/f*^*; NfatC1*^*Cre(+)*^) results in a statistically significant increase in activated, nuclear β-catenin (*P = .0002*) compared to control littermates at P0, as detected by a phospho-specific β-catenin antibody (pβ-catenin^S552^). This finding correlates with a significant increase in the β-catenin co-factor, Lef1 (*P < .01*), which is required for its transcriptional regulatory function. Previous reports have indicated that β-catenin activities may be dependent on the presence of primary cilia.^24,25^ As Dzip1 conditional knockout and mutant mitral valves were previously shown to have reduced cilia length,^14^ it is possible that increased β-catenin is due to loss of cilia, independent of a Dzip-Cby1 interaction. Analyses of β-catenin in cilia deficient (*NfatC1*^*Cre+*^*;Ift88*^*f/f*^) conditional knockout mitral valves failed to reveal a statistically significant change in activated β-catenin protein expression (Figure 4). These data indicate that the presence of cilia in the mitral valve does not directly affect activation of the β-catenin pathway.

### 2.5 | DZIP1 MVP mutation results in increased β-catenin signaling

**FIGURE 5:**
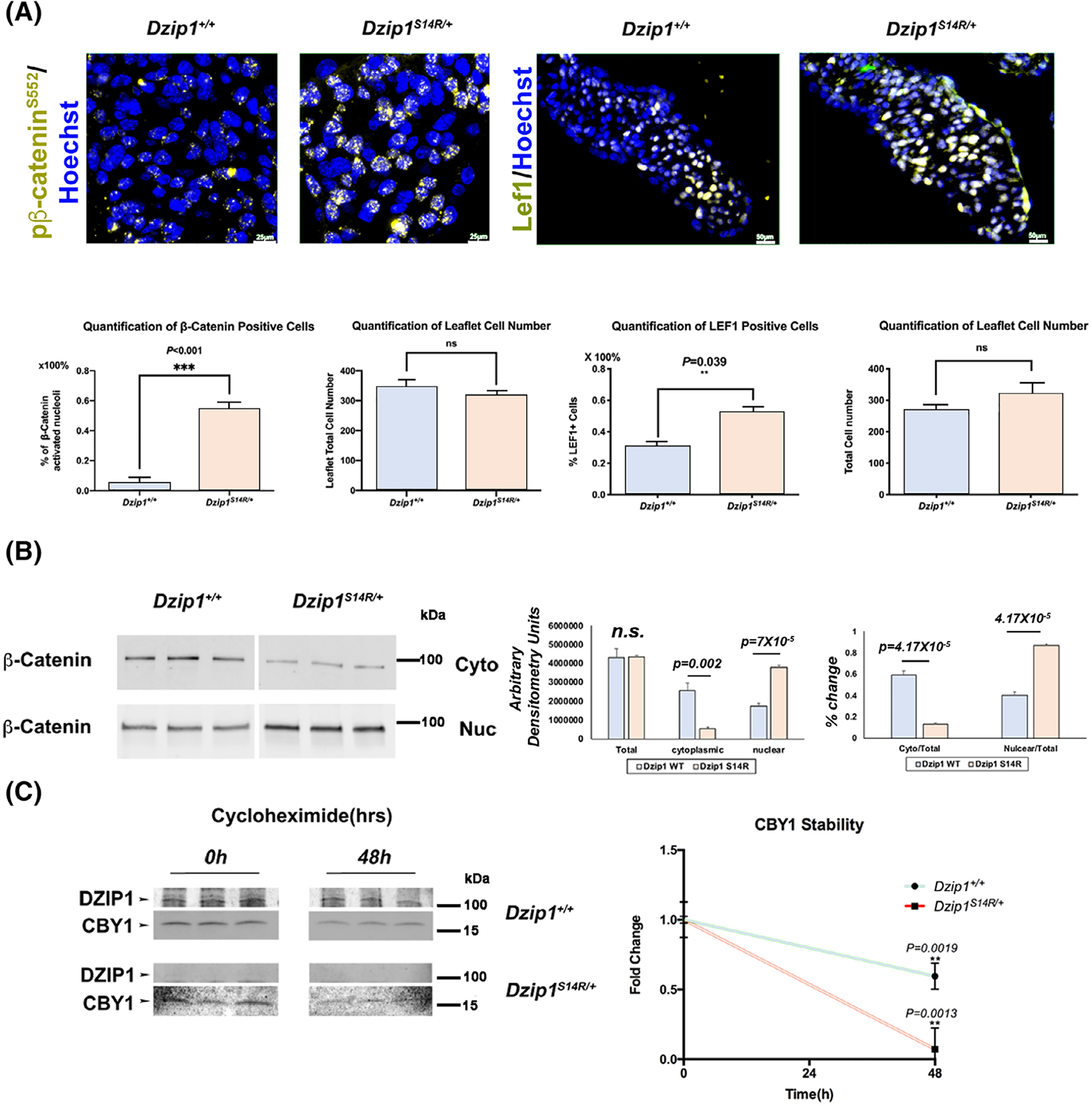
*Dzip1*^*S14R/+*^ causes increased β-catenin signaling. A, Left panel: IHC and quantification of pβ-catenin^S552 +^(gold) on *Dzip1*^*S14R/+*^ mitral valves at P0 compared to wild-type; right panel: IHC and quantification of Lef1^+^ (gold) cells on *Dzip1*^*S14R/+*^ mitral valves at P0 compared to wild-type. Nuclei were counterstained with hoechst (blue). Data are means ± SD, unpaired two-tailed Student’s *t*-test. (n = 3/genotype, ***P < .001, **P = .039, ns, non-significant). B, Western blots (left) and quantification (right) of β-catenin in E13.5 *Dzip1*^*S14R/+*^ mouse embryonic fibroblast (MEFs) nucleus and cytoplasm compared to wild-type. Data are means ± SD, unpaired two-tailed Student’s *t*-test.(n = 3/genotype, ns, non-significant). C, Western blots (left) and quantification (right) of cycloheximide experiment for CBY1 in E13.5 *Dzip1*^*S14R/+*^ MEFs compared to wild-type. Cby1 has a significant decrease in protein stability in the background of Dzip1 mutations. Data are means ± SD, two-tailed Student’s *t*-test. (n = 3/genotype)

Our previous data demonstrated that the *DZIP1*^*S24R/+*^ mutation found in MVP patients and its corresponding mutation in mice (*Dzip1*^*S14R/+*^) resulted in decreased protein stability and likely loss of function.^14^ Thus, we tested whether this loss of Dzip1 protein would result in a similar increase in nuclear β-catenin as observed in our conditional Dzip1 knockout mice (Figure 3). As shown in Figure 5, we observed a significant increase in activated nuclear β-catenin and its co-factor, Lef1 in the developing mitral valves. Subcellular fractionation of MEFs isolated from E13.5 *Dzip1*^*S14R/+*^ and wild-type littermates revealed a similar finding of increased nuclear β-catenin and reduced fraction of cytosolic β-catenin (Figure 5B). Total levels of β-catenin do not change in the MEFs nor do the total number of cells change in the valves. Coincident with this observation, cycloheximide experiments in *Dzip1*^*S14R/+*^ MEFs and wild-type controls revealed a significant reduction in Cby1 half-life when Dzip1^S14R^ was expressed (Figure 5C). Note that expression of Dzip1 is undetectable in our *Dzip1*^*S14R/+*^ MEFs even in the presence of a wild-type allele. This is similar to what we observed for the human mutation in immortalized patient lymphoblasts and indicates that the mutant protein has a detrimental effect on either the wild-type protein or wild-type allele.^14^

### 2.6 | Identification of a missense mutation affecting the CBY1 interaction domain in a family with autosomal dominant MVP

**FIGURE 6:**
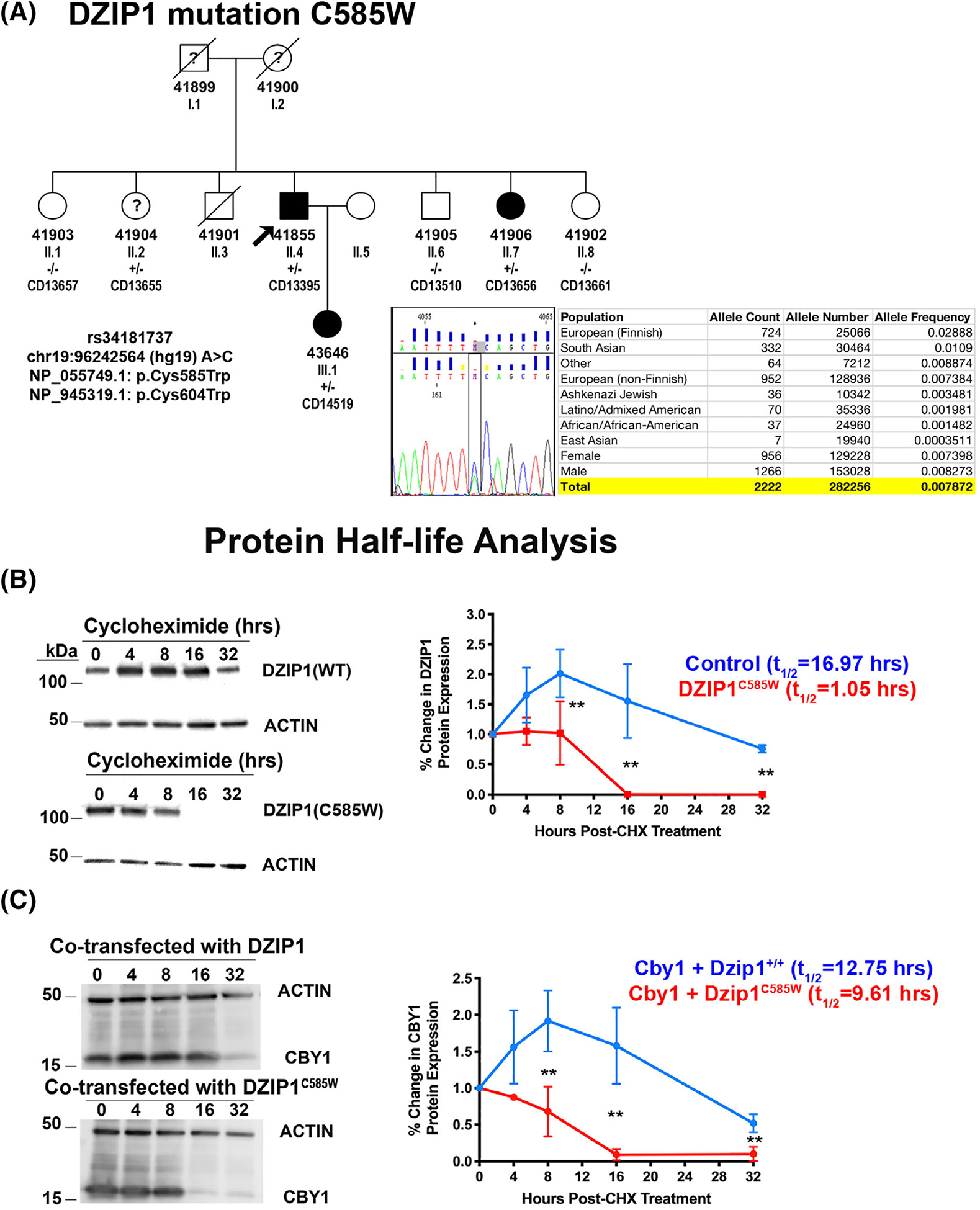
Identification of a DZIP1 missense mutation in a family with autosomal dominant MVP affecting the CBY1 interaction domain. A, Multigenerational family with inherited, autosomal dominant, non-syndromic MVP. Black circles and squares are affected individuals, and white circles and squares are unaffected. Diagonal circles and squares represent deceased individuals and were not available for the study. Circles, female; squares, male. ID designations for family members are denoted under the circles or squares. “?”, unknown phenotype. Proband is identified with the black arrow. Sanger sequencing identified a single missense mutation of DZIP1 (DZIP1^C585W^), resulting in a cysteine-to-tryptophan change. The mutation segregates with affected patients and is designated by “+/−” in the pedigree. Population frequency showing the rarity of the identified DZIP1 variant in the population. B, Western blots (left) and quantification (right) of cycloheximide (chx) experiment for DZIP1^C585W^ compared to DZIP1 control (WT). DZIP1^C585W^ has a significant decrease in protein half-life of 1.05 hours compared to 16.97 hours in wild-type (control). C, Western blots (left) and quantification (right) of cycloheximide experiment for CBY1 in presence of DZIP1^C585W^ compared to control. CBY1 protein has a significant decrease in protein half-life (9.61 hours) compared to control (12.75 hours). Data are means ± SD, unpaired two-tailed Student’s *t*-test. (n = 3, **P < .01)

Through our whole exome sequencing project of sporadic individuals and families with non-syndromic MVP, we identified a multigenerational family with a rare variant in DZIP1 *DZIP1*^*C585W/+*^ (Figure 6A). The mutation is rare with a minor allele frequency of 0.0078 and is predicted to be damaging by SIFT and Polyphen. This particular variant has a CADD (combined annotation dependent depletion) score of 22.5, which places it in the top 1% of deleterious single-base changes possible in the entire genome and within the 95% confidence interval of gene-specific CADD scores corresponding to high-confidence pathogenic mutations for DZIP1.^26^ The mutation segregates through two generations of the family and all affected individuals harbor the mutation. None of the confirmed non-MVP individuals have the mutation. Patient II.2 had indeterminant echocardiography and the status of her mitral valve was unable to be fully assessed. We tested mutation pathogenicity by performing cycloheximide experiments on transfected HEK293 cells and observed that the DZIP1^C585W^ mutation resulted in a decrease in protein stability with a reduced half-life from 16.97 hours to 1.05 hours (Figure 6B). To test whether the mutation also had an effect on CBY1 stability a similar experiment was performed in a separate CHX assay. As shown in Figure 6C, expression of DZIP1^C585W^ results in a premature loss of CBY1 expression consistent with our findings in the *Dzip1*^*S14R/+*^ mice.

### 2.7 | MVP DZIP1^C585W^ mutation alters protein structure and binding to β-catenin and CBY1

**FIGURE 7:**
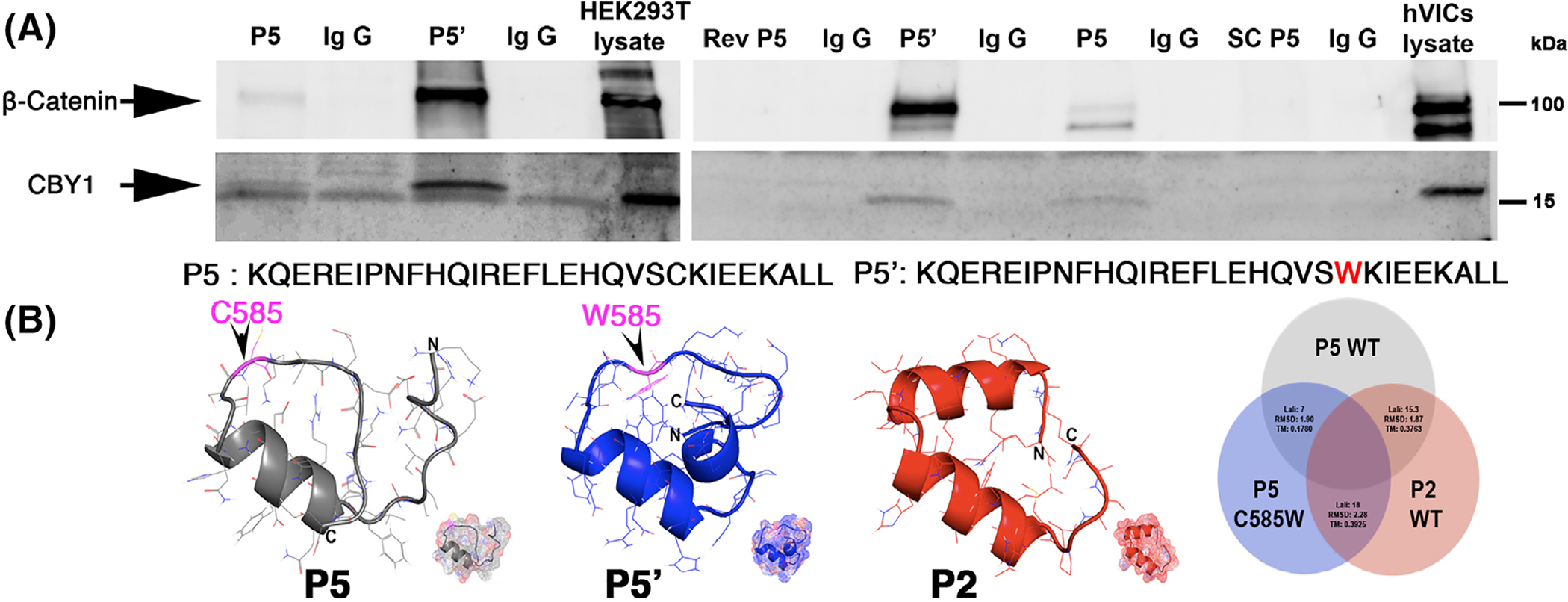
MVP DZIP1^C585W^ mutation alters protein structure and binding to β-catenin and Cby1. A, Co-IP and IB analysis of Peptide # 5 (P5) and mutant P5: P5’-C585W with CBY1 in HEK293T whole-cell lysis and cultured hVICs whole-cell lysis. P5’ = mutant P5 (C585W); Rev P5 = reversed P5; SC P5 = scrambled P5. P5’ binds with higher affinity than control (P5) peptide. B, Left: 3D modeling of P5, P5’, P2 showing increased similarity between P2 and P5’. Right: quantification of modeling data indicating P5 C585W mutant is more structurally similar to the P2 WT subunit compared to the P5 WT subunit

A peptide containing the C585W mutation was synthesized and is designated P5’ since its sequence is identical to that of the P5 peptide with the exception of the cysteine to tryptophan change. This peptide was tested for its ability to alter interactions with the CBY1-β-catenin complex. As shown in Figure 7A, both P5 and P5’ were capable of interacting with CBY1 and β-catenin. However, in all repeated experiments (N = 5), the P5’ mutant peptide demonstrated greater band intensity on the Co-IP reactions, suggesting an increased affinity for CBY1 and β-catenin. This result was confirmed in transfected HEK293 cell lines as well as primary mitral VICs during development. This result was strikingly similar to what we observed between P5 and the P2 segment, with the P2 peptide showing enhanced binding compared to P5 (Figure 2). Protein modeling and comparative structural predictions for each of the peptides was performed using RaptorX. Secondary and tertiary models for each of the peptides revealed a similar overall structure between P2 and P5’ (Figure 7B). Quantification of data obtained from structure modeling indicated that the cysteine to tryptophan (C585W) point mutation in the P5 subunit causes a drastic change in protein tertiary structure (TM Score = 0.1780). This modification renders the P5 C585W mutant more structurally similar to the P2 WT subunit (Lali: 18; RMSD: 2.28; TM Score: 0.3925) compared to the P5 WT subunit (Lali: 7; RMSD: 1.90; TM Score: 0.1780). Therefore, although the mutation causes faster degradation of the full-length protein, it is structurally more similar to the P2 region and confers increased binding to the CBY1 and β-catenin complex.

### 2.8 | β-catenin transcriptional responses are regulated through Dzip1 decoy peptides

**FIGURE 8:**
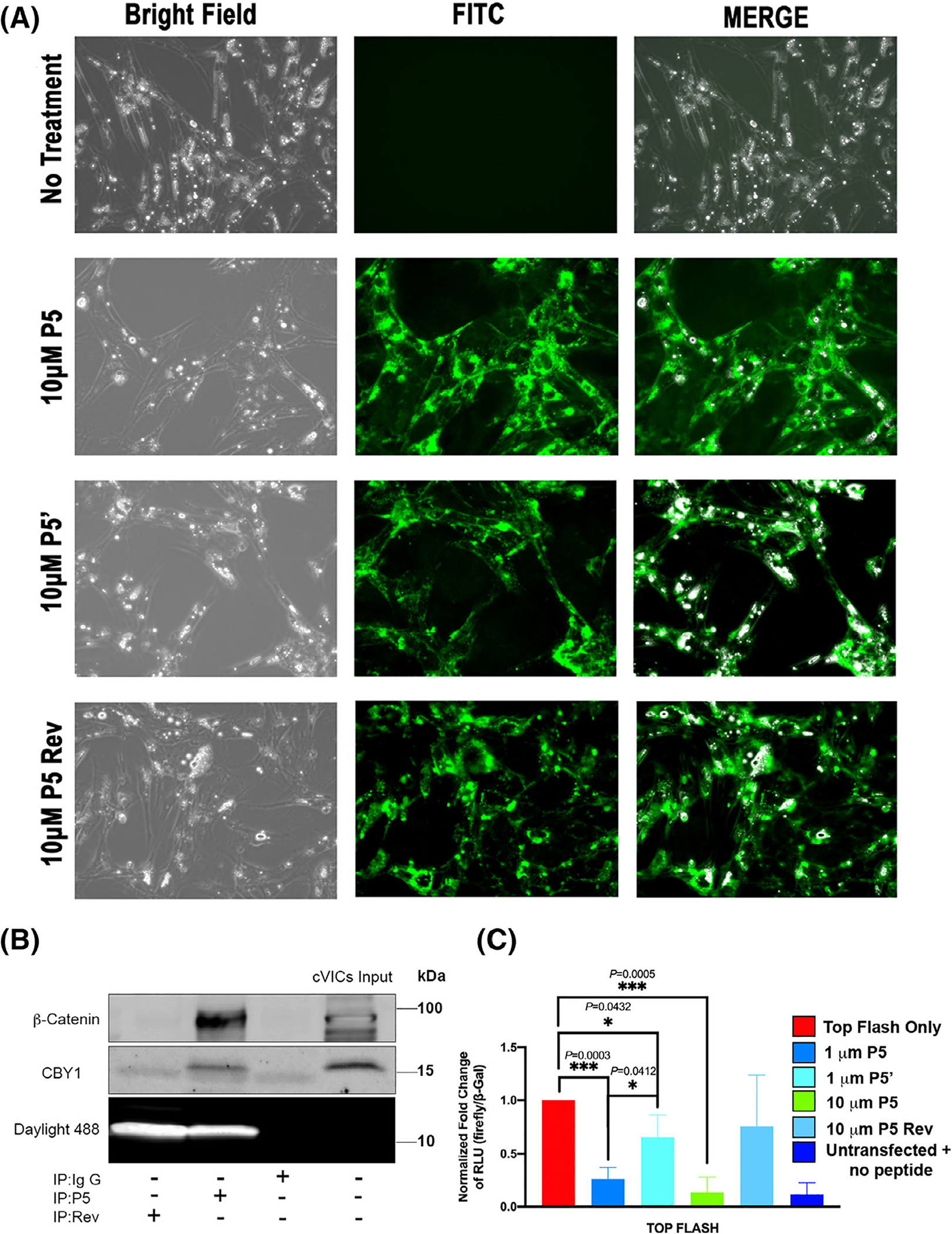
Dzip1 decoy peptides disrupts β-catenin transcriptional responses in vitro. A, Bright field and direct immunofluorescence of P5, P5’, and P5 Reverse (Rev) peptide treated chicken valvular interstitial cells (cVICs) showing membrane penetration of the peptides and overall normal cell morphologies. B, Co-IP and IB analysis of β-catenin/CBY1 with TAT conjugated P5 in cVICs whole-cell lysis showing that addition of the TAT sequence to the peptide did not have an effect on the ability for P5 to interact with CBY1 or β-catenin. Reverse (Rev) P5 was a negative control. TAT conjugated peptides on the gel were imaged under daylight 488. C, Luciferase assay of P5, P5’, Rev P5 peptide treated cVICs. β-Gal was used as an internal control

To determine whether transducing cells with these minimal interaction motifs could affect downstream β-catenin activities, we synthesized various peptides based on our Co-IP data and human DZIP1 mutation. Peptides for the P5 region as well as mutant P5 (P5’) and a P5 reverse peptide were synthesized with a cell-penetrating TAT peptide sequence and a 5-FAM moiety for fluorescent visualization at the amino terminus. Peptides were also biotinylated to be able to confirm interaction with CBY1 and β-catenin. These wild-type peptides were able to transverse the cell membrane (Figure 8A) and remained capable of interacting with both CBY1 and β-catenin (Figure 8B). The reverse peptide was unable to interact with Cby1 or β-catenin as expected. Cell viability and substrate attachment were not grossly affected with peptide administration. Localization of the peptides can be seen perinuclear and along the cell membrane. Presence of the peptides within the nucleus are not evident, suggesting potential retention of β-catenin outside of the nucleus.

To test whether the peptides function to disrupt β-catenin activities within the cell, VICs were transfected with the TOP flash β-catenin reporter plasmids. Forty-eight hours post-transfection, valve fibroblasts were stimulated with P5 (1 μM, 10 μM), P5’ (1μM) or reverse peptide (high dose 10 μM). Controls included cells that were transfected with the TOP flash reporter and no peptide as well as cells that were not transfected with DNA constructs or subjected to peptide treatment (untransfected + no peptide). Luciferase readouts from the TOP flash reporter confirmed a significant reduction in β-catenin activities when treated with both the P5 and P5’ mutant peptides at 1 μM dose (Figure 8B). P5 treatment at 10 μM dose resulted in a complete block in TOPFlash luciferase activation whereas the reverse peptide had no significant effect at this higher dose. Interestingly, the mutant P5’ peptide was not as robust in repressing the reporter as the wild-type, even though it showed enhanced interaction with the CBY1/β-catenin complex by biochemical approaches.

### 2.9 | Enhanced MMP2 expression and altered extracellular matrix deposition in *Dzip1*^*S14R/+*^ and *Cby1*^+/−^ postnatal mitral leaflets

**FIGURE 9:**
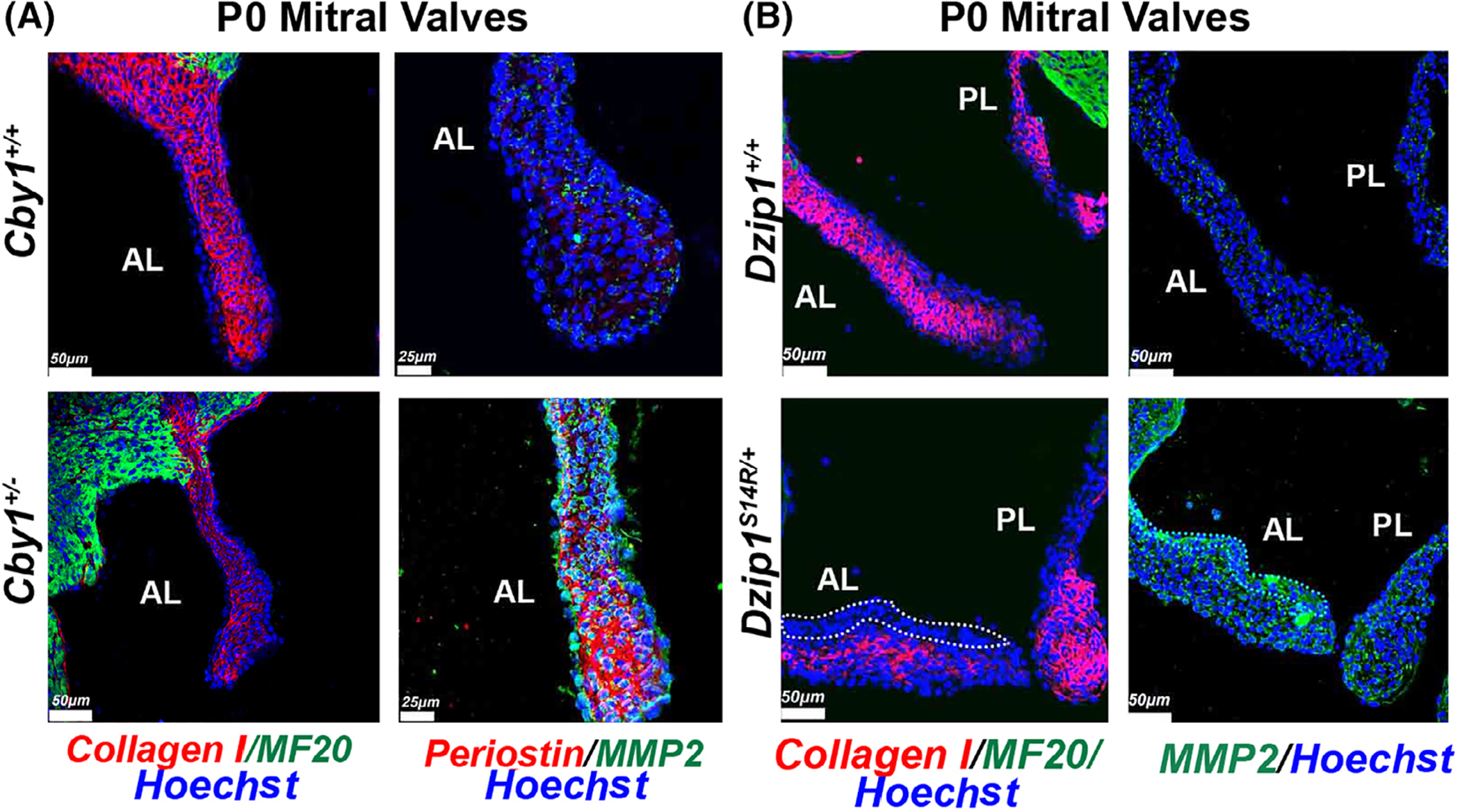
Enhanced MMP2 expression and altered extracellular matrix deposition in *Dzip1*^*S14R/+*^ and *Cby1*^+/−^ postnatal mitral leaflets. A, IHC of *Cby1*^+/−^ P0 anterior mitral leaflets for collagen I (red), MF20(green), periostin (red), MMP2 (green) and nuclei (Hoechst-blue) compared to wild-type (N = 3/genotype). B, IHC of *Dzip1*^*S14R/+*^ P0 anterior mitral leaflets for collagen I (red), MF20 (green), MMP2 (green) and nuclei (Hoechst-blue) compared to wild-type (N = 3/genotype). Dotted line highlights the MMP2 enriched area where collagen I deficient

Enhanced Wnt/β-catenin signaling has been observed in human myxomatous valves.^27–29^ Hyperactivated β-catenin can promote endothelial-to-mesenchyme transformation (EMT), endocardial proliferation, ECM remodeling and myxomatous degeneration of valves.^30–32^ In this study as well as our previous reports on Dzip1 mutations, we observe similar findings of increased β-catenin activities within the valves during development in a model (*Dzip1*^*S14R/+*^) that was previously validated as having myxomatous valves and MVP.^14^ For this study, we initially focused on MMP2 a known transcriptional target of β-catenin signaling^33^ that is known to be up-regulated in mitral valve disease.^7,8,34,35^ As shown in Figure 9, we observe increased MMP2 within the interstitium of Cby1 heterozygote (*Cby1*^+/−^) mitral valves by postnatal day 0. This finding of increased MMP2 was also observed in the *Dzip1*^*S14R/+*^ model of MVP. Although our previous RNAseq data^14^ showed an increase in collagen I mRNA at this time-point, IHC in both Cby1 and Dzip1 models show a loss of collagen protein within the valve. The reduction of collagen I, a known target of MMP2 enzyme activity was striking, especially along the atrialis of the mitral leaflets and is indicative of a potential increase in collagen fragmentation as is documented in human myxomatous valves. Interestingly, periostin, a known β-catenin transcriptional target^36^ is also up-regulated in the *Cby1*^+/−^ valves, which is consistent with findings in human myxomatous valves and in hyperactive β-catenin murine models.

## 3 | DISCUSSION

The genetic causes of mitral valve prolapse are only now beginning to be discovered. Once disease genes are identified, it will be important to identify the role that these genes play in disease initiation and progression. We recently identified mutations in the DZIP1 gene in multiple families with inherited non syndromic mitral valve prolapse.^14^ Through CRISPR-Cas9 genome editing, a murine model for non-syndromic MVP based on a human mutation was generated (*Dzip1*^*S14R/+*^). Utilizing a combination of proteomic based approaches with *in vivo* and *in vitro* studies, our findings reveal a mechanism by which myxomatous degeneration may occur through altered developmental pathways that invoke both ciliogenesis and β-catenin signaling. Previous studies have indicated that primary cilia can regulate canonical Wnt/β-catenin signaling.^14,24^ This is thought to occur through localization of Wnt receptors on the axoneme of the primary cilia and/or within the ciliary clefts. However, within cilia deficient mitral valves at P0, our studies did not reveal a significant change in activated nuclear β-catenin. Thus, our findings in Dzip1 models of increased β-catenin/Lef1 signaling highlights cilia-independent regulation of β-catenin pathways.

Our recent studies have highlighted a dynamic expression pattern between nuclear and membrane bound β-catenin.^27^ Whereas nuclear β-catenin is present during early embryonic development, a shift to the membrane is observed during fetal gestation and is maintained after birth. This likely highlights the multi-faceted role for this protein in both nuclear and membrane function. Mechanisms by which this nuclear vs cell membrane decision is made are not well understood, but likely critical for restricting β-catenin’s transcriptional function. Our studies highlight the unique possibility that β-catenin is held at the basal body through a Dzip1-Cby1 complex. Co-IP studies confirmed the formation of this complex and identified unique protein motifs that are critical for this interaction to occur. Generation of membrane permeant peptides of the Dzip1-Cby1 interaction motif potently suppressed β-catenin transcriptional regulation in vitro. However, it is unlikely that the peptides would confer sufficient structure to restrict the entire peptide-Cby1-β-catenin complex to the basal body. It is more likely that the interaction of the Dzip1-decoy peptide facilitates stabilization of a Cby1-β-catenin complex and inhibits nuclear shuttling independent of being sequestered at the basal body. This is further supported by our anecdotal evidence showing that loss of Dzip1 and/or mutations within the Dzip1-Cby1 interaction domain results in decreased protein stability concomitant with increased nuclear β-catenin and upregulation of transcriptional activities.

Whole exome sequencing identified a multigenerational family with a rare, damaging variant in the DZIP1-CBY1 interaction motif. Peptide analyses and Co-IP studies revealed that this mutation altered the 3D structure of the protein and conferred a paradoxical increase in Cby1-β-catenin interactions. This should theoretically result in an increase in the stability of the complex and a reduction in β-catenin activity. However, this is confounded by our data showing that the Dzip1^C585W^ mutation results in a profound reduction in protein half-life and premature degradation of CBY1 protein. Thus, although the DZIP1^C585W^ mutation appears to enhance an interaction with the complex, the reduced protein expression results in an overall loss of function, consistent with our other MVP-identified mutation, *DZIP1*^*S24R*^ and our *Dzip1* conditional knockout mice.

How altered β-catenin signaling may lead to a myxomatous valve and mitral valve prolapse is poorly understood. Previous reports have shown that either loss or gain of β-catenin function can result in a myxomatous phenotype.^30,32,37,38^ In the context of Dzip1 and Cby1, our data show that loss of either of these genes impairs ciliogenesis (Figure 1D and Toomer et al^14^). RNAseq and GO analyses by us and others have shown that loss of cilia during development results in transcriptional activation of numerous ECM genes including collagens and proteoglycans. In the context of increased collagens, one might expect to observe a fibrotic valve. Certainly, this occurs in the context of various other valve disease such as serotonin, carcinoid, or drug-induced (e.g., fenfluramine, ergotamine) valvulopathies where the valves show stiff “stuck-on plaques” and clear evidence of fibrosis. The phenotypes of these valves are very different from a floppy myxomatous valve, which is defined as having increased proteoglycans as well as fragmented collagen and elastin. This highlights that additional signaling pathways likely play a part in the fragmentation of collagen and the loss of properly stratified ECM boundaries. In this context, fragmentation of collagen would permit encroachment of proteoglycan proteins that can move via interstitial fluid flow within affected regions of the valve, particularly within the fibrosa. We believe our observation of increased MMP2 in our *Dzip1* and *Cby1* deficient mitral valves is significant since MMP2 is a direct target of β-catenin/Lef1 transcriptional regulation. Major substrates for this enzyme are collagens I, III, IV, V, VII, elastin, and TGF-β. Transcripts for each of these ECM genes are increased in both of our *Dzip1* and *Ift88* cilia deficient mice.^14^ However, there is an important discrepancy to note. The *Ift88* conditional knockout (*NfatC1*^*Cre(+)*^*; Ift88*^*f/f*^) mice show robust increases in collagen I transcript *and* protein at P0. The study presented in this report combined with our previous study paradoxically shows that that while *Dzip1*^*S14R/+*^ and *Dzip1* conditional knockout mice (*NfatC1*^*Cre(+)*^*; Dzip1*^*f/f*^) have increased transcripts for collagen and elastin genes, overall valvular collagen I protein levels are lower. A potential explanation for this finding is that in a mutated *Dzip1* tissue, increased β-catenin activity leads to upregulation of MMPs and subsequent proteolysis of collagens and elastin. Our study highlights that Dzip1 mutations result in a shift in the balance of ECM synthesis and destruction through loss of cilia and/or increased β-catenin signaling. This altered ECM homeostasis eventually leads to the generation of a myxomatous phenotype that is incompatible with normal valvular function. Thus, the molecular interaction between Dzip1 and Cby1 suppresses β-catenin signaling. This suppressive activity appears critical during development to ensure proper synthesis and organization of the valvular ECM.

## 4 | EXPERIMENTAL PROCEDURES

### 4.1 | Generation of Dzip1 expression constructs and TAT-fusion peptides

Control human DZIP1-Myc-DDK plasmid was purchased from Origene (clone ID: 198968). Truncated DZIP1 plasmids were sub-cloned with a HA tag sequence at the C terminus using ptarget^™^ Mammalian Expression Vector System (Promega, Cat No: A1410). The Dzip1 mutation was incorporated through a QuickChange II XL Site-Directed Mutagenesis Kit (Agilent Technologies) based on the manufacturer’s recommendations and had a HA-epitope tag at the C terminus. CBY1 plasmid with a HA-epitope tag at C terminus was purchased from Sino Biological (clone ID: BC016139). CBY1 plasmid with a Flag-epitope tag at the carboxy terminus was a kind gift from Dr.Ken-Ichi Takemaru and described previously.^21^ All clones were sequenced validated through Genewiz. TAT-fusion peptides were synthesized by Genescript with biotin at the carboxy-terminus and FAM at amino-terminus.

### 4.2 | Cell culture analyses

Human valvular cells (obtained from Dr. Adrian Chester, Imperial College London) were cultured in DMEM with 15% fetal calf serum and antibiotics(P/S, fungizone) as described previously.^11^ Chicken valvular interstitial cells (cVICs) were isolated from anterior leaflet of chicken embryos at HH40 as described previously.^39^ cVICs were maintained in Medium 199 (M199, Invitrogen) containing 5% of chicken serum (bio-world), 0.1% ITS (gibco,41 400-045) and 1% antibiotics(P/S, fungizone). *Dzip1*^*S14R/+*^ or wild-type mouse embryonic fibroblast(MEFs) were generated as previously detailed^40^ and were cultured in DMEM with 10% FBS and antibiotics (P/S, fungizone).For all experiments, valvular interstitial cells were utilized prior to passage 5. HEK293T cells were maintained in DMEM with 10% fetal bovine serum and antibiotics (P/S, fungizone).

### 4.3 | Mouse analyses and husbandry

*Dzip1* conditional mice and *Dzip1* knock in mice were genotyped and generated as previously described.^14^ *Cby1* mice were a kind gift from Dr. Ken-Ichi Takemaru. All animal experiments were performed under protocols approved by the Institutional Animal Care and Use Committees at the Medical University of South Carolina. Before cardiac resection, mice were euthanized by isoflurane (Piramal) induction, followed by cervical dislocation in accordance with the Guide for the Care and Use of Laboratory Animals (NIH publication no. 85-23, revised 1996). Comparisons of the data generated for both male and female sexes showed no appreciable differences. As such, combined data for both sexes are shown.

### 4.4 | Immunofluorescence

Immunohistochemistry (IHC) was performed as we have previously reported.^41^ Briefly, hearts from mice at post neonatal day 1 (P0) were fixed in 4% paraformaldehyde, embedded in paraffin, and sectioned at 5 μm. Deparaffinized sections were rehydrated through a graded series of ethanol’s to phosphate buffered saline (PBS; Sigma, St. Louis, Missouri). Sections were subjected to antigen unmasking (H-3300; Vector Laboratories, Burlingame, California) and treated for 1 hour at room temperature with a blocking buffer of PBS (Sigma, St. Louis, Missouri) containing 1% BSA (Sigma, B4287-25G). Primary antibodies used were mouse gamma tubulin (sigma, T6557,dilution 1:100), rabbit acetylated tubulin (cell signaling, 5335S,dilution 1:100), rabbit Arl13B(Protein tech, 17 711-1-AP, dilution 1:500), rabbit phospho β-catenin^S552^ (cell signaling,9566S,dilution 1:100). Primary antibodies were placed in blocking buffer overnight at 4°C. Following primary antibody incubations, specimens were washed five times in PBS and incubated at room temperature with Alexa Fluor goat α-rabbit 568 and goat α-mouse 488 (Invitrogen, Eugene, Oregon) diluted 1:100 in PBS. Nuclei were stained with Hoechst dye (1:10 000; Invitrogen) in PBS for 5 minutes prior to the final washes in PBS. All samples were cover-slipped using Dabco mounting medium (Sigma). Images of immunostained sections were captured with SP5 confocal microscopy (Leica Microsystems, Inc. Exton, Pennsylvania). Z-stacks were set by finding the highest and lowest depth with visible fluorescence and using the system optimized setting to determine steps. Z-stacks were then compiled to form maximum projection images.^13^ Files were transferred to Adobe Photoshop for labeling and figure preparation. For quantification of b-catenin levels through IHC, N = 3/genotype with six images captured from similar regions throughout the anterior mitral leaflets.

For measuring cilia length in the *Cby1* control (*Cby1*^+/+^) vs *Cby1*^+/−^, values were plotted every 0.5 μm to assess cilia length distribution differences between the two genotypes at P0. Three slides from a minimum of three animals/genotype were imaged at three different parts on anterior leaflets (tip, middle, and root). Cilia length measurements were performed using Z-stack images of anterior leaflet stained with acetylated alpha tubulin or arl13b, gamma tubulin and counterstained with Hoechst. Z-stack images were then imported into Imaris software and measurements were taken from the base to the tip of the axoneme. All cilia in the field of view were measured. For control mitral leaflets, a total of n = 1266 cilia lengths quantified. For *Cby1*^+/−^ mitral leaflets a total of n = 497 cilia lengths were quantified. Total cell numbers from each image were counted blindly by two people. n = 26 images were counted from wild-type and n = 25 images were counted from *Cby1*^+/−^.

### 4.5 | Co-IP and Western blotting

HEK293T cells were seeded at 5×10^5^ /well on 6-well plates 1 day before transfection. Cells were co-transfected with DZIP1 and CBY1 plasmids using Fugene HD transfection Reagent (Promega) for 72 hours and were lysed using ice-cold lysis buffer (25 mM Tris-HCL, 150 mM NaCl, 1% NP-40.5% glycerol) with Halt protease and phosphatase Inhibitor cocktail (×100) (Thermo Scientific, dilution 1:500) followed by incubating on ice for 30 minutes with intermittent vortexing. Preclearing of the cell lysates were done by adding 2.5 μL of IgG with the same species as primary antibody and 25 μL of Pierce Protein A/G Agarose beads (Thermo Scientific) into cell lysates containing 400 μg protein and rotating for 2 hours at 4°C. 5 μL of primary antibody or IgG together with 50 μL of Pierce Protein A/G Agarose beads were incubated with cell lysates at 4°C overnight. Beads were collected and washed with 1 mL of ice-cold lysis buffer for five times before SDS-PAGE. For peptide Co-IP, HEK293T or human valvular interstitial cell lysates were incubated with Pierce Streptavidin Magnetic Beads (Pierce, Inc.) and biotinylated peptide at 4°C overnight. Magnetic beads were washed, and complexes were eluted according to manufacturer’s recommendation prior to SDS-PAGE. Primary antibodies used for Co-IP assays and Western blotting were as follows: α-mouse Flag M2 (Sigma, dilution 1:1000), α-rabbit HA (Sigma, dilution 1:1000), α-rabbit CBY1(Protein tech, dilution 1:1000), α-rabbit β catenin (cell signaling, dilution 1:1000). Horse-radish peroxidase (HRP)-conjugated secondary antibodies were purchased from Sigma (dilution 1:10000). All Co-IP and western analyses were repeated a minimum of three times in independent biological replicates.

### 4.6 | Peptide treatment and reporter assay in cVICs

cVICs were seeded at 2×10^5^ /well on six-well plates. The next day, cells were transfected with 1.6 μg TopFlash (Addgene plasmid # 12456) or FopFlash (Addgene plasmid # 12457) and 0.4 μg β-galactosidase-encoding construct. Transfections were performed using FuGENE HD transfection Reagent (Promega) and cells were incubated at 37°C for 48 hours. TAT-fusion peptide was added at 10 μM or 1 μM 24 hours post transfection. Cells were harvested the next day after two washes of 1×PBS and firefly luciferase activities were evaluated using the Luciferase Assay System (Promega, E1500).

### 4.7 | Computational studies

RaptorX Structure Alignment was used to align the protein structures of interest: P5 wild-type (WT), P5 C585W mutant, P2 WT.^42,43^ RaptorX employs a statistical learning method to calculate the compatibility between a target sequence and a template structure.^44^ Protein structures were prepared using PEP-FOLD 3: a publicly available software capable of *de novo* peptide tertiary structure prediction from amino acid sequences.^45–47^ Protein structures were exported as PDB files and submitted for analysis by the RaptorX Structure Alignment Server. RaptorX calculates the structural similarity of two or more proteins by comparing the length of the core (Lali), root mean squared deviation (RMSD), and template modeling score (TM Score). For multiple structure alignment (MSA), Lali is the length of core, which consists of all the fully aligned columns.^42,43^ The RMSD is calculated only on the core residues. The TM score is the measure of similarity between two or more protein structures with different tertiary structures. The TM Score ranges from 0 to 1.0: If TM Score > 0.6, there is a 90% chance that two proteins share a similar fold. When TM Score < 0.4, there is a 90% chance that two proteins have different folds.

### 4.8 | Protein stability analysis

HEK293T cells were seeded at 5 × 10^5^/well on six-well plate. On the next day, cells were co-transfected with wild-type DZIP1-Flag or DZIP1^C585W^-Flag and CBY1-HA constructs using FuGENE HD transfection Reagent (Promega, Cat No: E2311). Cycloheximide was added 48 hours post transfection at concentration of 100 ng/mL. Cells were lysed with RIPA buffer at 0, 4, 8, 16, 32 hours after cycloheximide treatment. *Dzip1*^*S14R/+*^ and wild-type MEFs were seeded at 2 × 10^5^/well on six-well plate and treated with cycloheximide at concentration of 100 ng/mL the next day. Cell lysates were harvested using RIPA buffer at 0 and 48 hours post cycloheximide treatment. Immunoblotting was performed as described previously.^11^ Transfections were performed in triplicate and repeated a minimum of three times. Primary antibodies used for western blot were: α-mouse Flag M2 (Sigma), α-rabbit HA (Sigma), α-rabbit CBY1(Protein tech), α-rabbit DZIP1 (Protein tech), α-mouse actin (Millipore). HRP-conjugated secondary antibodies were purchased from Sigma.

### 4.9 | Nuclear and cytoplasmic extraction

*Dzip1*^*S14R/+*^ and wild-type MEFs from E13.5 embryos were plated at 2 × 10^5^ /well on six-well plates. On the following day, cells were released with trypsin and washed with 1× PBS twice and fragmentation was done following the instruction of NE-PER Nuclear and Cytoplasmic Extraction Reagents (Thermo, Cat NO:78835). N = 3/genotype. Nuclear and cytoplasmic extractions were collected for Western Blotting. β-Catenin was normalized by total protein loading by Ponceau S.

### 4.10 | Statistical analyses

All data are presented as means ± SD from two-tailed Students *t*-test.

### 4.11 | Human studies

All studies involving human research were approved by the Institutional Review Board Institut du Thorax, Nantes, France and all participants provided written informed consent.

### 4.12 | Familial genetics and WES

Exome sequencing was performed on the proband (II.4). Exome capture was carried out using the SureSelect Human All Exon System using the manufacturer’s protocol version 1.0 (Agilent Inc.) that is compatible with Illumina paired-end sequencing. Exome-enriched genomes were multiplexed by flow cell for 101-bp paired-end read sequencing according to the protocol for the HiSeq 2000 sequencer (version 1.7.0; Illumina) to allow a minimum coverage of 30×. Reads were aligned to the human reference genome (UCSC NCBI36/hg19) using the Burrows-Wheeler Aligner (version 0.5.9). Quality control to determine sample and genotyping quality and to potentially remove poor SNPs and/or samples was performed as we have previously published.^11^ Follow up Sanger sequencing was performed on the rest of the family to confirm phenotype-genotype correlation of the identified DZIP1 SNP.
